# Differing roles for short chain fatty acids and GPR43 agonism in the regulation of intestinal barrier function and immune responses

**DOI:** 10.1371/journal.pone.0180190

**Published:** 2017-07-20

**Authors:** Warren N. D’Souza, Jason Douangpanya, Sharon Mu, Peter Jaeckel, Ming Zhang, Joseph R. Maxwell, James B. Rottman, Katja Labitzke, Angela Willee, Holger Beckmann, Yingcai Wang, Yang Li, Ralf Schwandner, James A. Johnston, Jennifer E. Towne, Hailing Hsu

**Affiliations:** 1 Amgen Inc., Thousand Oaks, CA, United States of America; 2 Amgen Inc., Seattle, WA, United States of America; 3 Amgen Inc., Regensberg, Germany; 4 Amgen Inc., Cambridge, MA, United States of America; 5 Amgen Inc., South San Francisco, California, United States of America; Southern Illinois University School of Medicine, UNITED STATES

## Abstract

Inflammatory bowel disease (IBD) is associated with a loss of intestinal barrier function and dysregulated immune responses. It has been shown that short chain fatty acids (SCFAs) are protective in IBD and that GPR43 mediates the protective effects of SCFAs. In this study, we investigated the effects of SCFAs in comparison to highly specific GPR43 agonists on human intestinal epithelial and immune cells. Our results confirm that SCFAs are enhancers of barrier function in intestinal epithelial cells. Additionally, SCFAs also displayed potent immunoregulatory properties based upon the ability to inhibit LPS-induced cytokine production in PBMC, and human T cell proliferation and cytokine production. Unexpectedly, and in contrast to the current belief, specific GPR43 agonists failed to exhibit similar barrier enhancing and anti-inflammatory properties. These findings demonstrate that SCFA possess broad protective functions in IBD and agonizing GPR43 alone is unlikely to be beneficial in patients.

## Introduction

The human intestine harbors hundreds of trillions of bacteria that belong to thousands of different species. Under normal conditions, the microbiota share a symbiotic relationship with their host by aiding in carbohydrate digestion, vitamin synthesis, xenobiotic metabolism, strengthening of the intestinal barrier, providing protection from pathogenic infections, and the development of an appropriately regulated mucosal immune system. However, unhealthy changes in the microbial community (dysbiosis) are capable of resulting in diseases such as inflammatory bowel disease (IBD).

IBD is a condition wherein genetic and environmental factors converge to culminate in dysregulated immune responses in the gastrointestinal tract. Lesions are typically present in regions containing high bacterial concentrations, and there is considerable evidence that the immune response in IBD is directed towards, or driven by intestinal bacteria. For instance, increased cellular and humoral responsiveness to gut bacterial antigens is observed in IBD patients [1], disease symptoms in IBD models are reduced under germ-free conditions or following treatment with antibiotics [2], and changes in the intestinal microbiome composition have been shown to occur in IBD patients [3–7]. Uncontrolled immune responses to microbiota result in a compromised intestinal barrier and increased translocation of bacteria that trigger the immune system, thereby potentiating this cycle. In addition, impaired intestinal barrier function itself has been postulated to commence this cycle and initiate disease in IBD patients [8]. This notion of disease pathogenesis in IBD is supported by the finding that several genetic loci implicated in the disease include molecules involved in microbe recognition/defense, epithelial barrier function and innate/adaptive immune responses [9, 10].

Thus, it appears that a healthy microbiome maintains homeostasis in the intestine, but microbial dysbiosis is associated with dysregulated immune responses, compromised barrier function and disease. The mechanisms involved in these processes are only now being unraveled, and a group of molecules known to be involved in the cross-talk between the microbiome, the epithelium and the immune system are short chain fatty acids (SCFA). SCFA are carboxylic acids with aliphatic tails of less than 6 carbons (formic, acetic, propionic, butyric, isobutyric, valeric, isovaleric and caproic acids), that are produced in the intestine as products of anaerobic bacterial fermentation of unabsorbed carbohydrates and fiber [11]. Acetate, propionate and butyrate comprise >90% of the intestinal SCFA and concentrations of SCFA in the intestine can reach as high as 130 mmol/L [12]. SCFA are thought to serve as an energy source for intestinal epithelial cells, promote intestinal epithelial barrier function, and aid in the repair of wounded epithelium [13–18]. SCFA have also been shown to regulate immune cells [19–27], and ameliorate disease in rodent models of IBD [23, 24, 28–35]. Butyrate has also shown some promising effects in the treatment of ulcerative colitis [36]. However, the utility of this strategy was typically limited to distal UC due to the requirements for dosing via enema.

Elucidation of the molecular mechanisms responsible for the protective effects of SCFA in IBD can provide novel targets that are more amenable to a traditional drug development approach, and thus to optimal dosing for maximal efficacy. In this regard, SCFA were shown to bind to, and signal through GPR41 (FFA3/FFAR3), GPR43 (FFA2/FFAR2) [37–39], and GPR109a [40], and recent evidence points to a direct and indispensable role for GPR43 in mediating the protective roles of SCFA in IBD. For example, GPR43 deficient mice were reported to develop exacerbated disease in mouse models of colitis; and SCFA were reported to ameliorate colitis in WT, but not GPR43 deficient mice [25, 34, 35]. Herein, we investigated the role for SCFA in maintaining intestinal barrier integrity and immune cell function in human cells, and tested whether GPR43 agonists were capable of eliciting similar responses as SCFA. Our results reveal that SCFA were potent enhancers of intestinal barrier function and inhibitors of immune cell activation; but GPR43 agonists were incapable of substituting for SCFA.

## Materials and methods

This study was approved by the Amgen Institutional Animal Care and Use Committee (approval number 2009–00002), and Amgen Institutional Review Board approval and consent was obtained for collection and the studies involving human blood.

### Cell lines, intestinal epithelial cell isolation and droplet digital PCR (ddPCR)

C2BBe1 [clone of Caco-2] (CRL-2102™), HT-29 (HTB-38™), T84 (CCL-248™), Caco-2 [Caco2] (HTB-37™) were obtained from ATCC. Primary mouse intestinal epithelial cells were isolated using EDTA treatment of intestinal pieces followed by a percoll gradient. Cells were stained with EpCAM and CD45 and analyzed using flow cytometry to confirm that the majority of the cells isolated were epithelial cells. Cells were lysed in Qiagen RNeasy lysis buffer (RLT) and frozen until processing. RNA was purified with an RNeasy kit (Qiagen) and cDNA was synthesized using SuperScript III (Invitrogen). GPR43 primers were purchased from Invitrogen (mouse-Mm02620654_s1, human-Hs00271142_s1) and ddPCR were performed in triplicate using Bio-Rad QX100 Droplet Digital PCR system, according to the manufacturer’s instructions.

### cAMP inhibition assay

A competitive, HTRF® immunoassay (CisBio Dynamic 2 cAMP kit) was used to measure the inhibition of cAMP response in CHO cells stably expressing human GPR43. Assay-ready cells were thawed, resuspended in PBS and stimulated with forskolin (3μM final) for 45 minutes at room temperature in the presence of serially diluted natural ligands or test compounds in a 384 well plate (Greiner). Subsequently the cells were lysed by adding lysis buffer containing the cAMP-Tracer and Europium-labeled anti-cAMP antibody according to the manufacturer’s protocol. The signals were recorded using a Perkin Elmer Envision. The raw data was calculated as fluorescence ratio (665nm/620nm) and analyzed using GraphPad Prism Software.

### Aequorin assay

Agonist-induced receptor activation was determined by an increase in cytosolic calcium concentration in CHO-K1 cells stably expressing the mitochondrial targeted aequorin and human FFA2 receptor. The increased cytosolic calcium was monitored by luminescence emitted from aequorin as previously described [41]

### Intestinal barrier function measurements using TEER and Lucifer Yellow

C2BBe1 cells were cultured in the apical chamber of transwells (Corning) coated with type I collagen (BD Biosciences) in DMEM containing 10% FBS for one day prior to switching the cells to DMEM containing 1x MITO Extender (BD Biosciences) instead of FBS for an additional 4 days. TEER was measured using a REMS Autosampler (World Precision Instruments). For Lucifer yellow measurements, the cells were switched to assay buffer (HBSS/HEPES) on day 4, and 100 μM Lucifer Yellow was added to the apical chamber of transwells. The next day (day 5), TEER measurements and presence of Lucifer yellow in the basal chamber of the transwells was measured using a Perkin Elmer Envision.

### Intestinal barrier function measurements using ECIS

C2BBe1 cells were cultured in the apical chamber of transwells (Corning) coated with type 1 collagen (BD Biosciences) and placed in the ECIS transwell array. Cells were cultured in DMEM containing 10% FBS, and the next day (day 1), the medium was replaced with that containing 0.5% serum. On day 2, media was changed and test agents were added in duplicate in media containing 0.5% serum. The experiment was concluded 48 hours later (day 4). Barrier resistance was measured on the ECIS instrument. The plots shown are at 75 Hz beginning at day 2 post-addition of the test agents with resistance values normalized to those observed just prior to the addition of test agents. For barrier disruption and reformation studies, cells were cultured in transwells in the ECIS array as described above in the presence of 10% serum for one day and 0.5% serum for another day. On day 2, the media was replaced with media containing 0.5% serum and 2.5 mM EGTA (in apical and basolateral chambers). The EGTA was washed off 5 hours later and replaced with media containing 0.5% serum and test agents. Cells were monitored over the next 48 hours for barrier reformation and the plots shown are at 75 Hz with resistance values normalized to those observed just prior to the addition of test agents.

### LPS stimulation of PBMC

Amgen Institutional Review Board approval and consent was obtained for collection and the studies involving human blood. PBMC were isolated from heparinized blood using Ficoll and then plated onto 96-well plates. Butyrate or GPR43 agonists were incubated with the PBMC for 45 minutes prior to the addition of 1 μg/mL lipopolysaccharide (LPS). Cell culture supernatants were collected at 18 hours post-stimulation. TNFα, IL-1β and IL-6 production were measured using Meso Scale Discovery multiplex kits.

### Mixed lymphocyte reaction

Amgen Institutional Review Board approval and consent was obtained for collection and the studies involving human blood. PBMC were obtained from volunteers’ human blood using Ficoll. T cells were isolated using magnetic sorting with a pan T cell isolation kit (Miltenyi Biotec). Stimulator cells were isolated by negative magnetic sorting using anti-CD3 beads to deplete CD3+ cells. The stimulator cells were irradiated and mixed together with the purified T cells in the presence or absence of the SCFA or agonist. Supernatants were collected at day 3 for cytokine measurements using Meso Scale Discovery multiplex kits, and proliferation was measured at day 4 after an 18 hour pulse with 1μCi 3H-thymidine per well.

## Results

### Measuring the potency of GPR43 agonists

**Fig 1:**
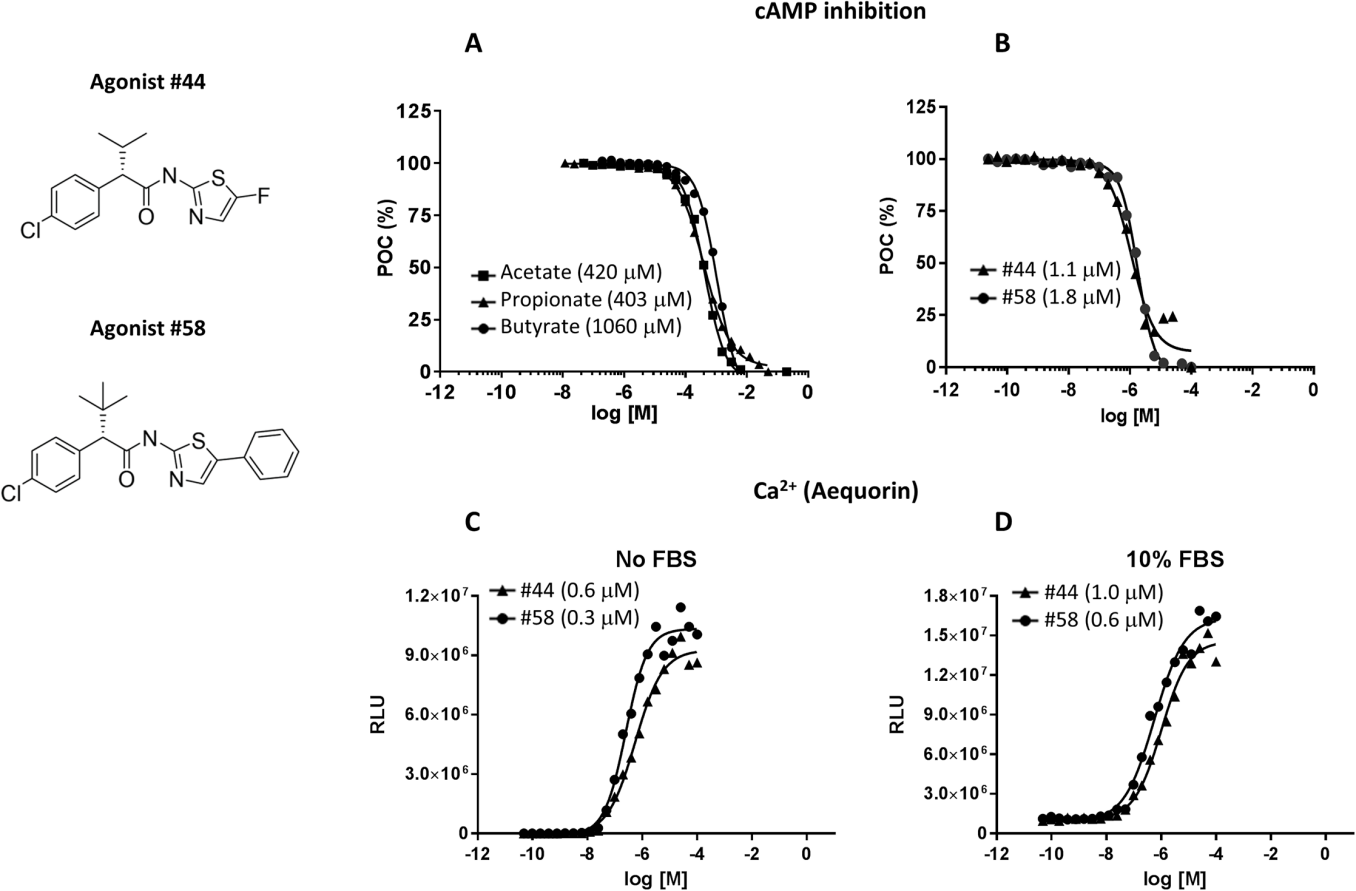
Potency of GPR43 agonists. (A, B) Inhibition of forskolin-induced cAMP production in CHO cells stably expressing human GPR43 was measured in the presence of the SCFA (A) or GPR43 agonists (B). The data was plotted as percent of control (POC), to normalize to the levels of cAMP obtained following stimulation with forskolin alone. (C, D) GPR43 agonist-induced receptor activation in CHO-K1 cells stably expressing human GPR43 was determined by measuring the increase in cytosolic calcium concentrations. This was performed with the indicated GPR43 agonists in the absence (C) or presence (D) of 10% FBS. Plots represent the average, and the results shown are representative of 3 independent experiments.

SCFA signaling through GPR43 results in the inhibition of cAMP production and the induction of calcium mobilization *via* dual coupling to Gα_i_ and Gα_q_ G proteins, respectively [37–39]. Consistent with these reports, acetate, propionate and butyrate, the three major intestinal SCFA, activated GPR43 and led to the inhibition of forskolin-induced cAMP production in CHO cells stably expressing human GPR43 (Fig 1A; EC_50_ values indicated in parenthesis). As reported previously, SCFA demonstrated relatively low potency (EC_50_ in the sub-millimolar to low single digit millimolar range) and the relative potency order was propionate≥acetate≥butyrate. We have previously reported on the identification of allosteric agonists for GPR43 that exhibited greatly increased potency than the endogenous ligands, and were inactive against GPR41, GPR109A and GPR40 [41, 42]. Similar to the endogenous SCFA ligands, these agonists are capable of GPR43-mediated inhibition of forskolin-induced cAMP production as well as calcium mobilization. The phenylacetamide agonists #44 and #58 from Wang, Y. et al., were chosen and their activity on cAMP inhibition (Fig 1B) and calcium mobilization (Fig 1C) was confirmed using a GPR43 transfected cell line. Although the Gα_i_ and Gα_q_ pathways operate independently downstream of GPR43, it is important to note that these GPR43 agonists displayed a similar range of activity on both signaling arms. We wished to profile these agonists *in vitro* using media that included serum, and measured the potency of the agonists in the presence of 10% FBS. All compounds potently inhibited forskolin-induced cAMP production in the presence of 10% FBS with only a 2-fold decrease in potency compared to serum-free conditions (Fig 1D). Thus, these compounds provided us with the tools to determine the effects of GPR43 agonism on human cells *in vitro* in the presence of serum. Given their increased potency (EC_50_ values in the sub-micromolar to low single digit micromolar range), micromolar concentrations of the GPR43 agonists were compared to millimolar concentrations of SCFAs in all subsequent experiments.

### Expression of GPR43 in intestinal epithelial cells

**Fig 2:**
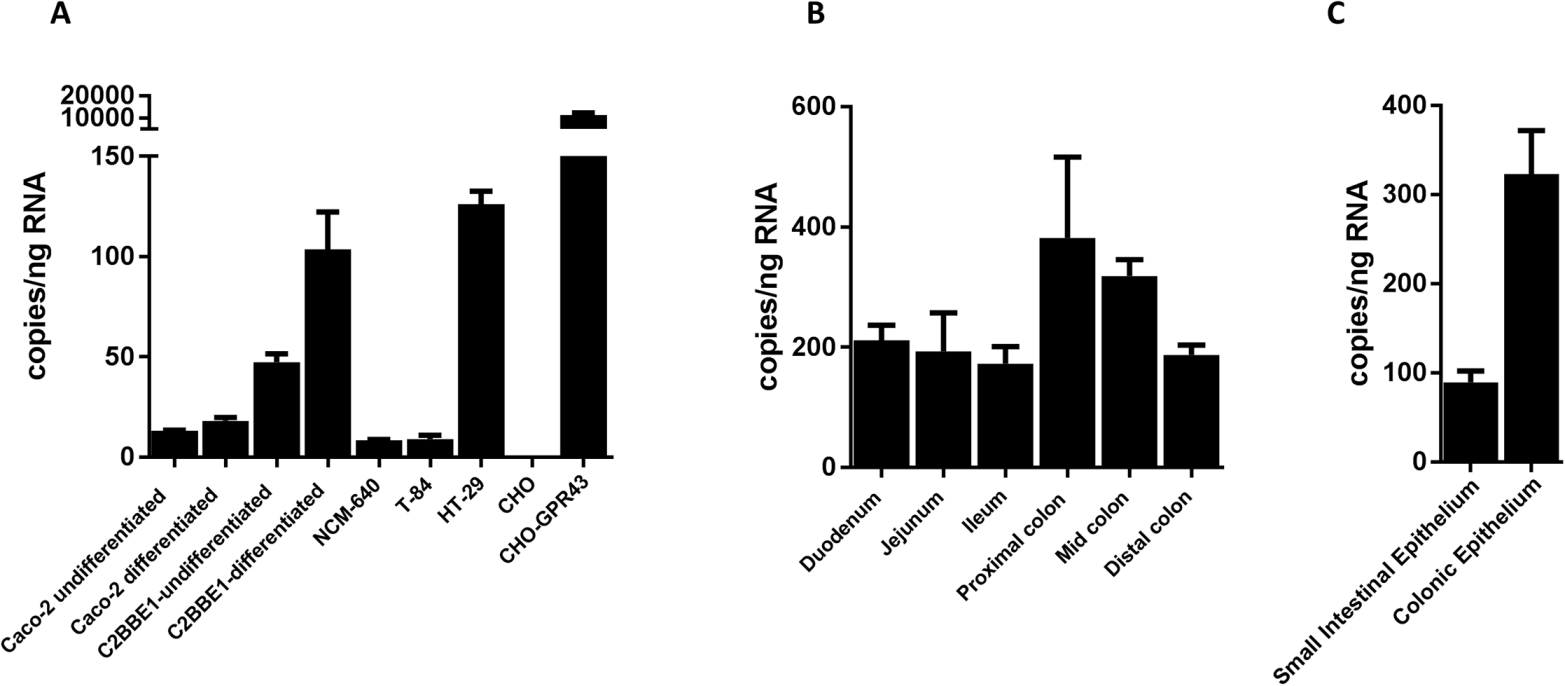
Expression of GPR43 in intestinal cells. (A) The expression of GPR43 mRNA in the indicated cell lines was measured using droplet digital PCR (ddPCR). Parental CHO cells and cells transfected with GPR43 served as the negative and positive controls, respectively. (B, C) GPR43 expression in different regions of the mouse intestinal tissue (B) or purified intestinal epithelial cells (C) was measured using ddPCR. Results were expressed as copies of GPR43 per ng of isolated RNA. Plots represent the average of duplicates/triplicates with standard deviation.

Impaired epithelial barrier function in the intestine is thought to play a key role in the progression of IBD, and one of the mechanisms whereby SCFA are thought to mediate their therapeutic activity in IBD is by enhancing barrier function. The highest levels of GPR43 expression are found on neutrophils, but GPR43 has also been shown to be expressed in the gastrointestinal tract [37–39]. We wished to determine the role of GPR43 in the intestinal epithelium, and examined GPR43 expression in human intestinal epithelial cells using droplet digital PCR (ddPCR). GPR43 was expressed at low levels on Caco-2, NCM-640 and T-84 cells, but appreciable levels of expression were observed in HT-29 and the C2BBe1 clone of Caco-2 cells (Fig 2A). GPR43 expression was also observed in mouse intestinal tissue (Fig 2B) and expression in the epithelial compartment was confirmed by using purified intestinal epithelial cells (Fig 2C). It has been reported that SCFA levels are higher in the colon compared to the small intestine [12] and we observed a similar finding for the SCFA receptor GPR43. RNA isolated from CHO cells transfected with human GPR43, and parental CHO cells was used as a positive and negative control, respectively.

### Enhancement of intestinal epithelial barrier function by butyrate

**Fig 3:**
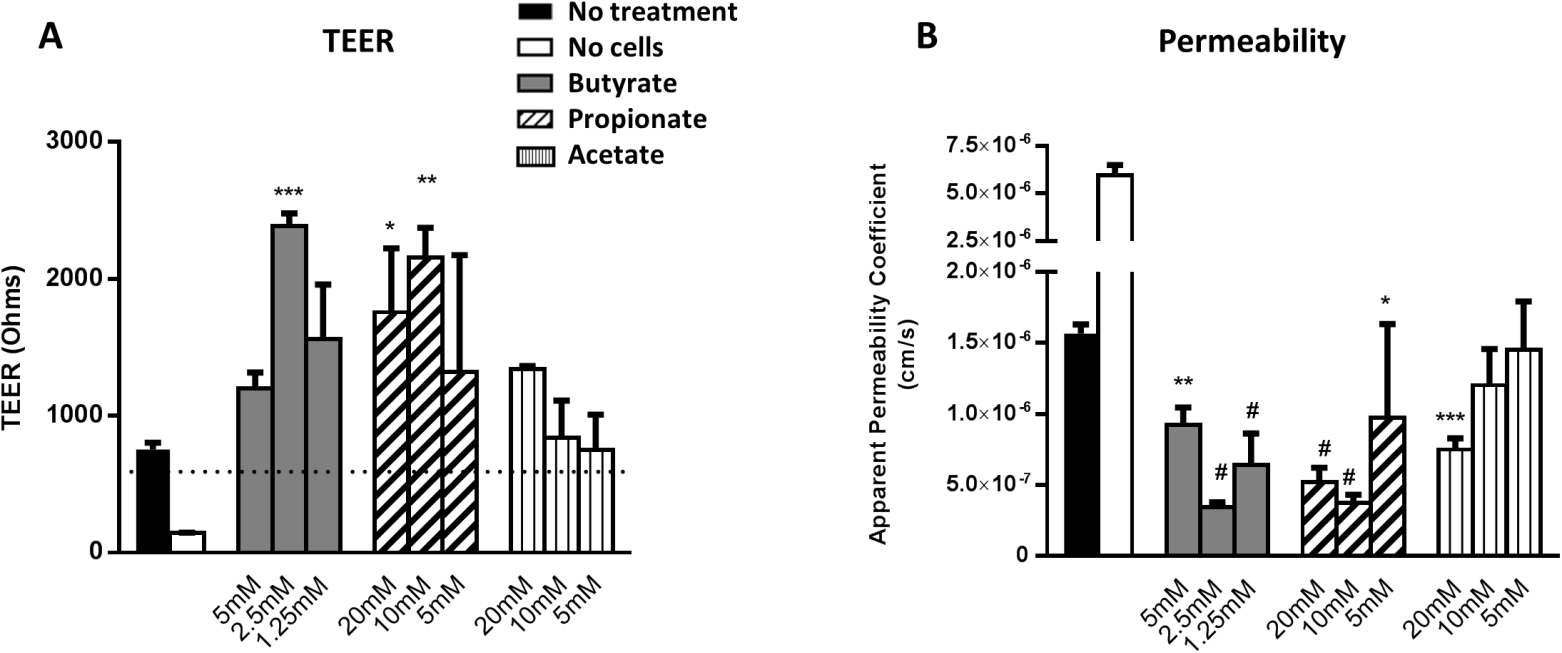
Enhancement of TEER by butyrate. C2BBe1 cells were cultured in the apical chamber of transwells in the presence of the indicated SCFA. TEER measurements were taken every day and on day 4, assay buffer containing Lucifer Yellow was added to the apical chamber of transwells. (A) Results of the TEER measurements on day 5 were plotted. (C) Lucifer Yellow levels in the basal chamber on day 5 were plotted. Plots represent the average of duplicates/triplicates with standard deviation, and the results shown are representative of 2 independent experiments. Statistics were calculated using ANOVA with Dunnett’s MCT comparing all treatment conditions to the ‘no treatment’ control (*P ≤ 0.05, **P ≤ 0.01, ***P ≤ 0.001, # P ≤ 0.0001).

Based upon GPR43 expression, the C2BBe1 clone of Caco-2 cells were chosen to determine the effects of SCFA and GPR43 agonists on intestinal barrier function. We began by testing the effects of different concentrations of acetate, propionate and butyrate on transepithelial electrical resistance (TEER), a measure of barrier function, using monolayers of C2BBe1 cells. Based upon published data [13, 15] suggesting that butyrate was toxic to intestinal epithelial cells at concentrations >5 mM, lower concentrations of butyrate were used compared to acetate and propionate. In agreement with published reports, 5 mM of butyrate also appeared to be toxic in our assays. Nonetheless, all 3 SCFA tested were capable of enhancing TEER in intestinal epithelial cells (Fig 3A). The increased TEER readings obtained following SCFA treatment correlated very well with a reduction in paracellular permeability to Lucifer yellow (Fig 3B). Butyrate stood out as the most potent of the three SCFA at enhancing intestinal epithelial barrier function. This was interesting given that butyrate displayed the least potency of the three SCFA on GPR43 in GPCR signaling assays (Fig 1). This observation did suggest that butyrate may be functioning in a GPR43-independent manner (see discussion).

**Fig 4:**
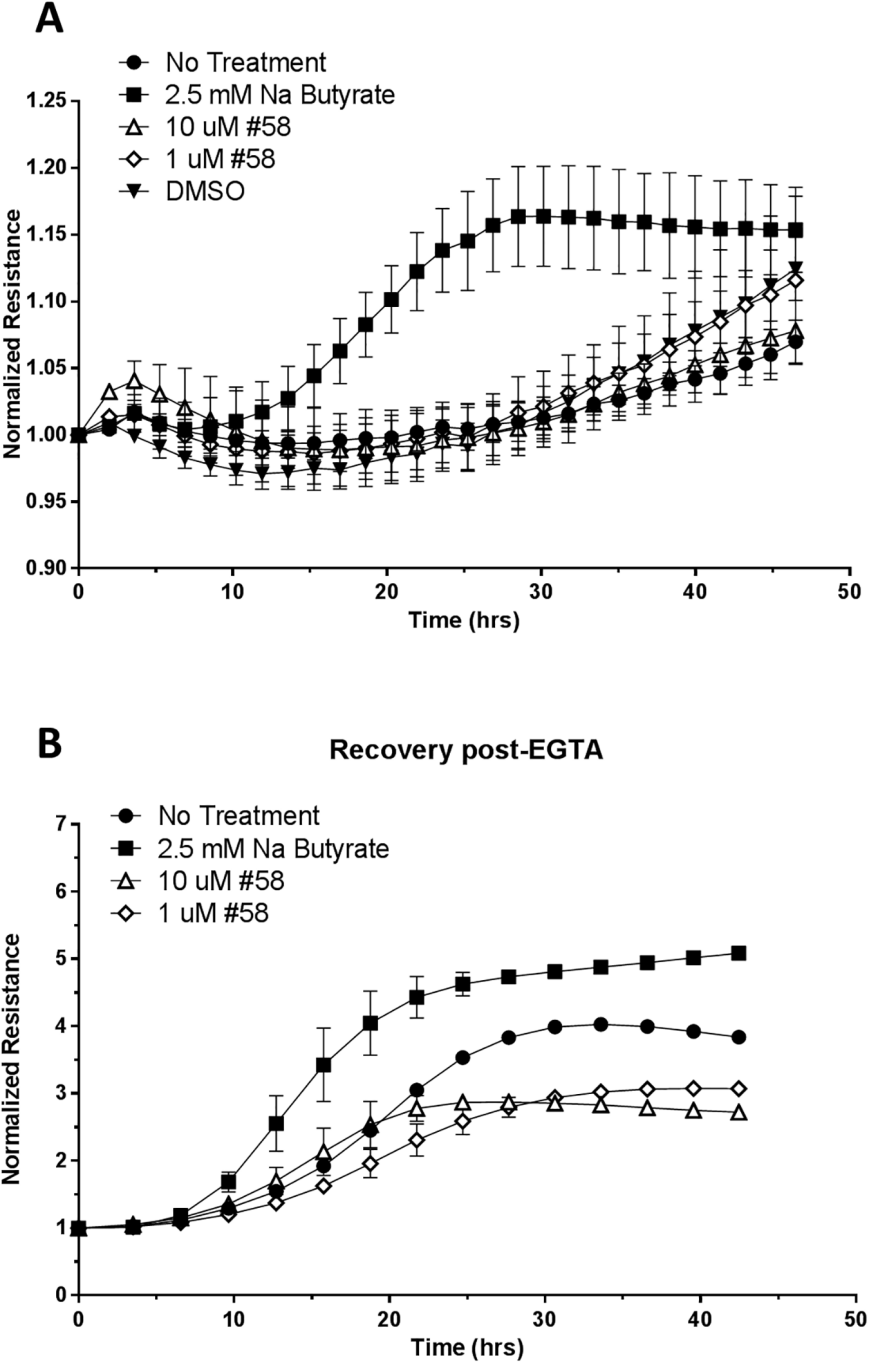
Enhancement of intestinal epithelial barrier function by butyrate. (A) C2BBe1 cells were cultured in the apical chamber of transwells in the ECIS transwell array. Barrier resistance was measured on the ECIS instrument @ 75 Hz beginning at day 2 post-addition of the test agents with resistance values normalized to those observed just prior to the addition of test agents. (B) Cells were cultured as in A for 2 days. The media was then replaced with media containing 2.5 mM EGTA (both apical and basolateral chambers) for 5 hours prior to washing and replacing with media containing test agents. Cells were monitored over the next 48 hours for barrier reformation and the plots shown are at 75 Hz with resistance values normalized to those observed just prior to the addition of test agents. The results shown are representative of 2 independent experiments.

To investigate further and compare the effects of butyrate to GPR43 agonists, we chose to use an ECIS instrument (electric cell-substrate impedance sensing) to obtain a more dynamic measurement of barrier function over time, rather than just a static measurement at single time-points. Butyrate led to a quicker increase, as well as higher measurements of resistance in C2BBe1 cells compared to untreated cells, suggesting more rapid formation of a tigher barrier (Fig 4A). We then determined if the effects exhibited by butyrate could be mimicked by GPR43 agonism by testing the effects of 1 uM and 10 uM agonist #58 in the same assay. However, this was not the case, as the use of even the the high concentration of the GPR43 agonist did not result in increased resistance when compared to the DMSO control (Fig 4A). The differentiation of intestinal epithelial cells *in vitro* has been shown to lead to a concurrent increase in resistance measurements and we wondered if the barrier enhancing effects of butyrate were merely due to effects on C2BBe1 cell differentiation. In order to test this hypothesis, cells were allowed to differentiate and form a barrier in the absence of any treatment, and the barrier was then disrupted by treatment with EGTA. The EGTA was washed off a few hours later, butyrate or the GPR43 agonist was added in, and barrier reformation was measured over time. As expected, loss of resistance was observed following treatment with EGTA, but resistance then increased over time following removal of EGTA (Fig 4B). This demonstrated that cell death was not responsible for the decrease in resistance with EGTA treatment. Upon observing barrier reformation in this study, butyrate once again exhibited potent barrier promoting properties, and led to earlier and higher levels of resistance. In contrast, the GPR43 agonists did not increase barrier resistance, and in fact appeared to lead to slightly lower values. Thus, it was clear that short chain fatty acids like butyrate in particular were strong enhancers of barrier function in intestinal epithelial cells, but GPR43 agonism was unable to mimic these effects on the epithelial barrier.

### Anti-inflammatory effects of butyrate on LPS-induced cytokine production

**Fig 5:**
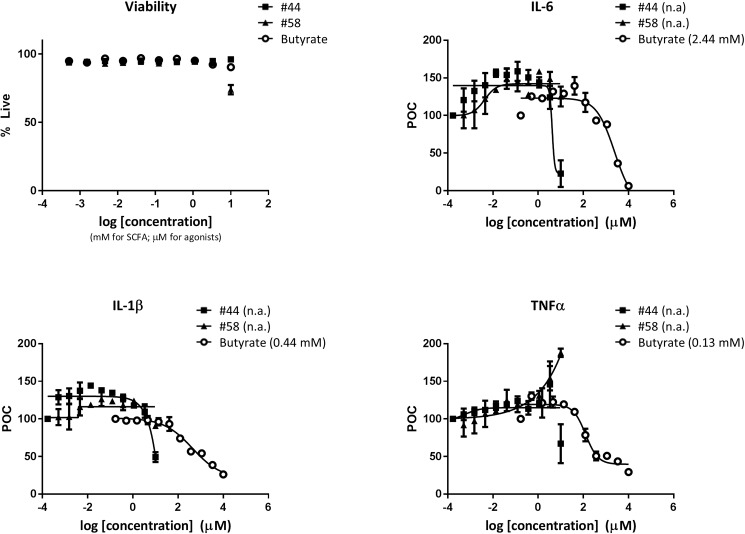
Anti-inflammatory effects of butyrate on LPS-induced cytokine production. Human PBMC were incubated with the indicated concentrations of butyrate (mM range) or GPR43 agonists (μM range) for 45 minutes prior to the addition of 1 μg/mL LPS. The levels of TNFα, IL-1β and IL-6 production in the supernatants were measured 18 hours later. Inhibition curves were plotted and IC_50_ concentrations are indicated in parenthesis when applicable. For the panel displaying cell viability, the concentrations of SCFA in mM and agonists in μM were plotted along the same axis for easier viewing. The data plotted are the values obtained following the subtraction of background levels of cytokines obtained without LPS stimulation and represent the average of duplicates with standard deviation. The results shown are representative of 2 independent experiments consisting of a total of 4 donors.

As mentioned earlier, inappropriate activation of immune cells by bacterial components is thought to be a key driver of IBD pathogenesis, and it has been demonstrated that SCFA are capable of inhibiting pro-inflammatory cytokine production induced by bacterial LPS [20, 21, 43]. Human PBMC express GPR43 [37–39] and hence, we tested the ability of butyrate and GPR43 agonists to inhibit cytokine production following a short-term stimulation of human PBMC with LPS. As shown in Fig 5, butyrate potently inhibited the production of LPS-induced IL-1β and TNFα. Butyrate also inhibited IL-6 production, albeit with reduced potency. The inhibition of pro-inflammatory cytokine production in the presence of butyrate was not due to toxic effects on the cells since cell viability was not altered even at the highest concentrations of butyrate utilized in this assay. In contrast to the potent effects of butyrate on LPS-induced cytokine production, only the highest concentration of the agonist #44 resulted in inhibition of cytokine production while agonist #58 might have in fact led to a slight increase in some cytokines. The data plotted are the values obtained following the subtraction of background levels of cytokines obtained without LPS stimulation. Thus, there was again a marked dichotomy when comparing the effects of butyrate with those of GPR43 agonists on the inhibition of proinflammatory cytokine production by human PBMC.

### Anti-inflammatory effects of butyrate in a mixed lymphocyte reaction

**Fig 6:**
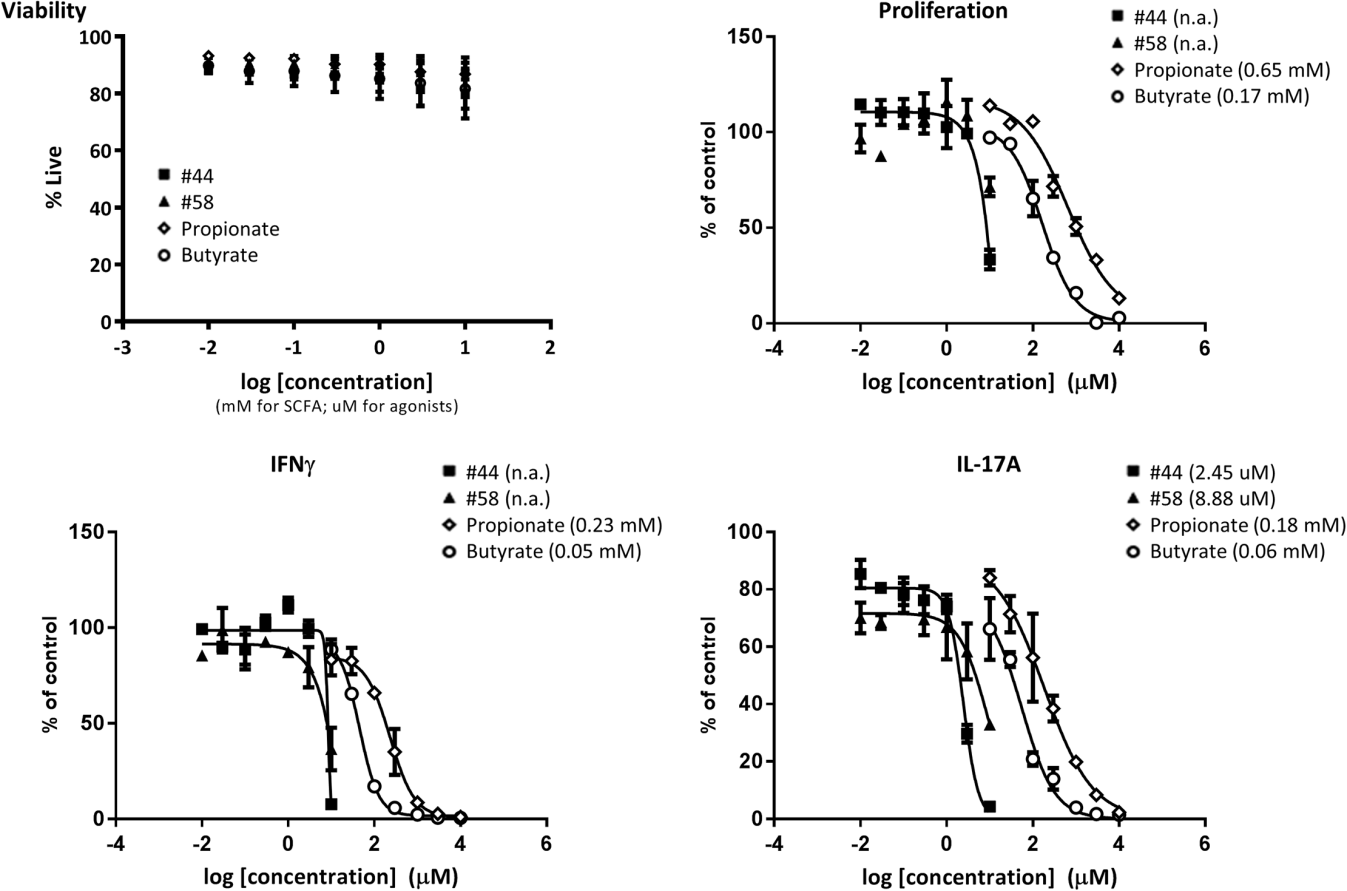
Anti-inflammatory effects of butyrate in a mixed lymphocyte reaction. Purified human T cells were stimulated with CD3-negative irradiated PBMC in the presence or absence of indicated SCFA or agonists. Cytokines in the supernatant were measured at day 3 post-activation, while viability and proliferation was measured at day 4. Inhibition curves were plotted and IC_50_ concentrations are indicated in parenthesis when applicable. For the panel displaying cell viability, the concentrations of SCFA in mM and agonists in μM were plotted along the same axis for easier viewing. Plots represent the average of duplicates with standard deviation, and the results shown are representative of 2 independent experiments consisting of a total of 4 donors.

It is well established that T cells are involved in the pathogenesis of IBD, and for this reason, we tested the effects of SCFA and GPR43 agonists on human T cell proliferation and cytokine production in a one-way mixed lymphocyte reaction. T cells isolated from human PBMC were cultured with irradiated allogeneic stimulator cells in the absence or presence of propionate, butyrate or two GPR43 agonists. Cytokine levels in the supernatants were measured at day 3 post-activation while proliferation was measured at day 4 following an overnight pulse with ^3^H-thymidine. Activated T cell proliferation was inhibited in a dose-dependent manner following treatment with butyrate and propionate (Fig 6). These SCFA were extremely potent at inhibiting the production of effector cytokines such as IFNγ and IL-17, which are known to play a role in IBD. Measurement of the proportion of live cells by flow cytometry confirmed that these results were not attributed to non-specific effects on cell viability. It is important to note that the effects of butyrate were seen at concentrations lower than the EC_50_ values observed for butyrate activity on GPR43 (see Fig 1). In contrast to the effects observed with SCFA, only the highest concentrations of GPR43 agonists inhibited proliferation or IFNγ production. Interestingly, the agonists were capable of inhibiting IL-17 production, but yet again the results obtained using GPR43 agonists contrasted dramatically with those obtained using SCFA. These data highlight the broader role that we observed for SCFA, apart from their role as ligands for GPR43.

## Discussion

Short chain fatty acids play a key role in intestinal homeostasis and are capable of protecting against dysregulated immune responses in the intestine. However, given the myriad of functions ascribed to SCFA, it is unclear which of these serve as the major protective mechanisms in IBD. The finding that GPR43 KO mice displayed exacerbated disease in colitis models [34, 35] suggested that GPR43 may mediate the protective effects of SCFA in intestinal inflammation. However, this data is controversial as other groups have observed reduced inflammation in the absence of GPR43 under certain experimental conditions [44, 45] and our studies revealed no difference when comparing disease severity in WT and GPR43 KO mice during DSS-induced colitis (S1 Fig). Nonetheless, the demonstration that the protective effects of SCFA in IBD models were abrogated in the absence of GPR43 provided strong evidence that GPR43 mediated the protective effects of SCFA during intestinal inflammation in these specific models [25, 34, 35].

Given these findings, GPR43 seemed a likely candidate mediating the barrier-enhancing and anti-inflammatory properties of SCFA in IBD, and modulation of GPR43 is being investigated as a novel therapeutic strategy in the treatment of IBD. Hence, we wished to test this hypothesis directly by comparing and contrasting the effects of SCFA and specific GPR43 agonists on human intestinal and immune cells. Our results demonstrated a prominent role for SCFA as enhancers of intestinal barrier function and inhibitors of immune cell activation. However, GPR43 agonists were incapable of substituting for SCFA in the assays employed.

The signaling pathways utilized by SCFA appear to be dependent upon the cell type and the function being examined. For example, cell surface GPCRs have been proposed to mediate SCFA-induced increases in intestinal epithelial barrier function [16], and SCFA promote cytokine and chemokine production by intestinal epithelial cells via GPR41 and GPR43 [44]. Within the immune system, SCFA induce mouse neutrophil chemotaxis via GPR43 [46]; and regulate allergic inflammation via GPR41, but not GPR43 [47]. In contrast, it appears that SCFA regulate neutrophil apoptosis [48], PBMC and macrophage cytokine production [21, 43], and effector/regulatory T cell differentiation [23, 24, 49], via HDAC inhibition. Lastly, to add to the complexity of this system, GPR109a on immune and intestinal epithelial cells has been shown to play a role in butyrate-mediated protection against colonic inflammation and carcinogenesis [50]. Thus, it is very likely that some of these GPR43-independent mechanisms are operational in our assays, and we think it is unlikely that GPR43 agonism alone will provide the same benefit as SCFA in the treatment of IBD. However, it would be of great interest to determine if any of the other pathways operating downstream of SCFA can be harnessed as novel therapeutic strategies.

## Supporting information

S1 Fig GPR43 deficiency did not alter the development of DSS colitis. (A) WT C57BL/6 or GPR43 deficient mice were adminstered 3% DSS and weight and clinical scores measured daily over the course of a week. Data represent the average score ± SEM from 2 mice (water) or 8 mice (DSS) per group. (B) Histological scoring of distal colon tissue taken from mice on day 7. Each point represents an individual mouse. The solid line represents the group mean. Scores are on a 0–5 scale.
(PDF)
