# Supplementary material for: Differing roles for short chain fatty acids and GPR43 agonism in the regulation of intestinal barrier function and immune responses
Source: PLoS One. 2017 Jul 20;12(7):e0180190. doi: 10.1371/journal.pone.0180190 (PMC5519041; doi:10.1371/journal.pone.0180190)
Supplement: S1 Fig — (A) WT C57BL/6 or GPR43 deficient mice were adminstered 3% DSS and weight and clinical scores measured daily over the course of a week. Data represent the average score ± SEM from 2 mice (water) or 8 mice (DSS) per group. (B) Histological scoring of distal colon tissue taken from mice on day 7. Each point represents an individual mouse. The solid line represents the group mean. Scores are on a 0–5 scale. (PDF) [file pone.0180190.s001.pdf]

S1 Fig

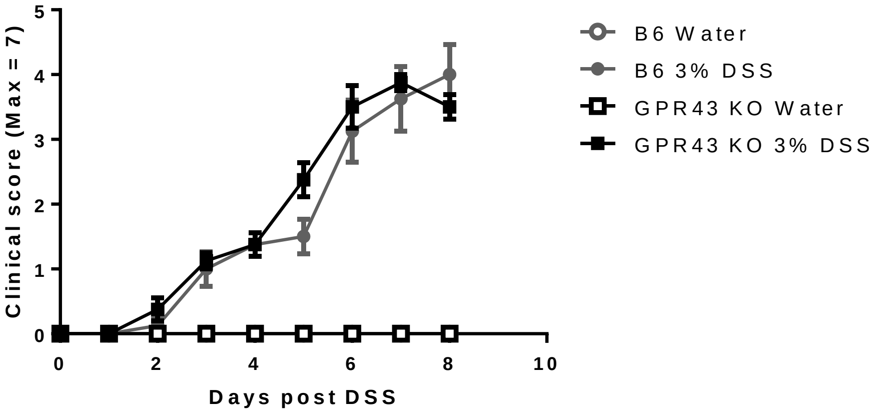

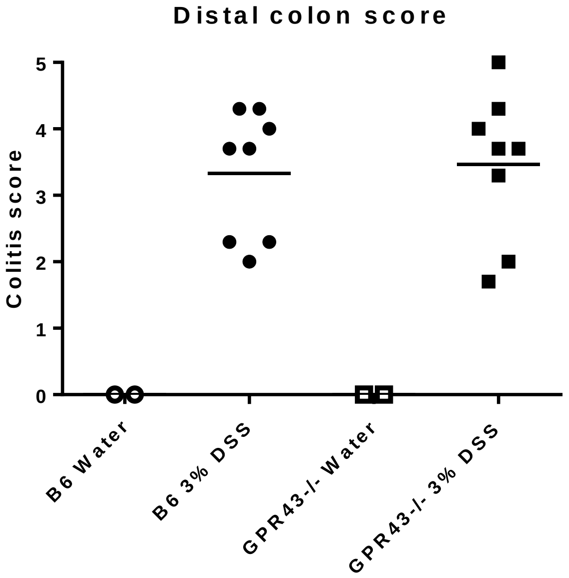

FIGURE S1. GPR43 deficiency did not alter the development of DSS colitis. WT C57BL/6 or GPR43 (Ffar2) deficient mice were administered 3% DSS and weight and clinical scores measured daily over the course of a week. (A) Body weights and clinical scores were monitored daily throughout the study. Clinical score was graded 0-3 for stool consistency, 0-2 for blood in stool, and 0-2 for mouse appearance, for a total possible score of 7. Data represent the average score ± SEM from 2 mice (water) or 8 mice (DSS) per group. (B) Mice were euthanized on day 8, and the distal colon was collected, cut into 3 cross sections for H&E staining, and scored on a 0-5 scale by a pathologist. Each point represents an individual mouse and the solid line represents the group mean.

D'Souza et al.

Supplementary Methods

Female C57BL/6 mice and GPR43 (Ffar2) deficient mice were obtained from Taconic. Colitis was induced by adding 3% DSS (w/v) to the drinking water for 7 days. On day 7, the DSS was replaced by regular drinking water. Body weights and clinical scores were monitored daily throughout the study. Clinical score was graded 0-3 for stool consistency, 0-2 for blood in stool, and 0-2 for mouse appearance, for a total possible score of 7. Mice were euthanized on day 8 by carbon dioxide asphyxiation followed by cervical dislocation. The distal colon was collected, cut into 3 cross sections for H&E staining, and scored on a 0-5 scale by a pathologist.
